# 

**Published:** 1906-08-18

**Authors:** 


					the hospital
TALf
rRnhanrintinn Terms"! wainffinaiftiA*
^ " ^4fv?wiwr7 rrco Tcnn I fmrinMJLCLi
go. 1,039. %ol. XL. [rgyX"l Satubday>
August 18th, 1906.
[ aee p. 356. rma] PR1CE THREEPENCE

				

